# Facilitating access to voluntary and community services for patients with psychosocial problems: a before-after evaluation

**DOI:** 10.1186/1471-2296-9-27

**Published:** 2008-05-07

**Authors:** Justin Grayer, John Cape, Lisa Orpwood, Judy Leibowitz, Marta Buszewicz

**Affiliations:** 1 Centre for Outcomes Research and Effectiveness (CORE), Sub-department of Clinical Health Psychology, University College London, 1-19 Torrington Place, London, WC1E 7HB, UK; 2 Camden and Islington NHS Foundation Trust, St Pancras Hospital, 4 St Pancras Way, London, NW1 0PE, UK; 3 Primary Care Mental Health Development Team, Islington PCT, 338-346 Goswell Road, London, EC1V 7LQ, UK; 4 Primary Care Mental Health Development Team, Camden PCT, Public Health Department, St Pancras Hospital, 4 St Pancras Way, London, NW1 0PE, UK; 5 Royal Free & University College Medical School, Research Department of Primary Care and Population Health, 2nd floor, Holborn Union Building, Archway Campus, Highgate Hill, London, N19 5LW, UK

## Abstract

**Background:**

Patients with psychosocial problems may benefit from a variety of community, educational, recreational and voluntary sector resources, but GPs often under-refer to these through lack of knowledge and time. This study evaluated the acceptability and effectiveness of graduate primary care mental health workers (GPCMHWs) facilitating access to voluntary and community sector services for patients with psychosocial problems.

**Methods:**

Patients with psychosocial problems from 13 general practices in London were referred to a GPCMHW Community Link scheme providing information and support to access voluntary and community resources. Patient satisfaction, mental health and social outcomes, and use of primary care resources, were evaluated.

**Results:**

108 patients consented to take part in the study. At three-month follow-up, 63 (58%) had made contact with a community service identified as suitable for their needs. Most were satisfied with the help provided by the GPCMHW in identifying and supporting access to a suitable service. There was a reduction in the number of patients with a probable mental health problem on the GHQ-12 from 83% to 52% (difference 31% (95% CI, 17% – 44%). Social adjustment improved and frequencies of primary care consultations and of prescription of psychotropic medications were reduced.

**Conclusion:**

Graduates with limited training in mental health and no prior knowledge of local community resources can help patients with psychosocial problems access voluntary and community services, and patients value such a scheme. There was some evidence of effectiveness in reducing psychosocial and mental health problems.

## Background

Many patients present with psychosocial problems in primary care [1]. Whilst some may be helped by referral to counselling or mental health services, others can potentially benefit from a variety of community, educational, recreational and voluntary sector resources. However, it can often be difficult for primary care teams to help people access these resources appropriately, due to lack of knowledge about what is available and insufficient time to facilitate this access [2-5].

Schemes where GPs refer patients to a link worker with knowledge of community organisations can improve access of patients to community and voluntary sector resources [5-7]. A qualitative evaluation of a 'social prescribing' scheme in South London found patients reported a reduction in isolation and an increase in self-esteem [5]. In a randomised control trial of a 'referral facilitation' scheme in Bristol, referred patients had improved mental health outcomes [7].

In previous studies, the link worker has had extensive pre-existing knowledge of the local voluntary and community sectors and often also significant training and experience in health and social care [5-7]. This limits the widespread adoption of such schemes, as individuals with such detailed local knowledge and health training are likely to be scarce or expensive. The present study aimed to evaluate the feasibility of such a role being undertaken in primary care settings by a graduate primary care mental health worker (GPCMHW) with limited previous training in mental health, and no previous knowledge of local community resources [8]. Use of GPCMHWs would allow for widespread adoption of such schemes. The study evaluates the acceptability to patients and effectiveness of a GPCMHW in this role.

## Methods

### Design and setting

A before-after design was used. GP practices in the two inner-city London Boroughs of Camden and Islington were contacted by letter and email. Thirteen practices volunteered and participated in the study over a one year period. In one Borough a 'hub and spoke' model was adopted, whereby the GPCMHW was based at four practices (for half a day/week each), but accepted referrals from an additional two local surgeries. In the second Borough the GPCMHW was based for a half-day a week or fortnight at each of seven GP practices.

### Patients

Patients 18 years old or over with a psychosocial problem were referred by members of the primary health care team to the GPCMHW. Definition of psychosocial problems was broad to allow referrers latitude to refer any patient they thought might benefit from the Community Link service, and included common mental health problems such as anxiety and depression, and social problems such as isolation, relationship, housing and financial difficulties which might impact negatively upon patients' psychological wellbeing. Exclusion criteria were active suicidal ideation, current episode of acute psychosis or crisis, being housebound, requiring a specialist mental health service or already being under the care of secondary mental health services or social services (this last criterion as the service funding was specifically for patients not under care of specialist mental health services). At the initial appointment a verbal and written explanation of the study was provided and patients signed a consent form agreeing to future contact and access to their medical notes by a research assistant. If a patient declined to take part in the research study, it was made clear that they were still eligible to access the service.

### Intervention

During the initial appointment the GPCMHW carried out a semi-structured assessment of the patient's psychosocial needs and administered the study baseline questionnaires. In this or a subsequent appointment, the GPCMHW researched and advised the patient about potential community resources which might help meet their identified needs; the GPCMHW utilised a combination of paper and electronic directories, telephone enquiries, and other sources. When required, the GPCMHW supported the patient's attendance at recommended organisations, for example, making contact with or accompanying the patient to their initial meeting with the organisation. Information about assessments and action were communicated to the referrer and documented in the patient's primary care medical record. A more detailed description of the service can be found elsewhere [9].

Three months after the initial assessment with the GPCMHW, a research assistant met with the patient to administer the study follow-up measures. If the patient did not want a face-to-face follow up appointment or defaulted, the questionnaires were posted.

The GPCMHWs were two recent psychology graduates who had some previous clinical experience in a voluntary capacity, but had no formal mental health training. Once in post the workers received, in-house training and ongoing supervision from two clinical psychologists.

### Measures

#### General Health Questionnaire-12 (GHQ-12) [10]

Measures mental or emotional distress, on a 12-item 4-point scale scored 0 0 1 1. The standard clinical threshold of 2(+) was used.

#### Clinical Outcomes in Routine Evaluation-Outcomes Measure (CORE-OM) [11]

Measures global distress on a 34-item Likert scale ranging from 0 to 4. The standard clinical threshold score of 10 was used.

#### Work and Social Adjustment Scale (WSAS) [12]

Measures the impact of the patient's problem on work, home management, social and private leisure activities, and relationships, utilising a 6-item Likert scale ranging from 0 to 8.

#### Client Satisfaction Questionnaire (CSQ) [13]

Measures general satisfaction with amount, effectiveness and quality of the service used by the respondent on an 8-item Likert scale ranging from 1 to 4.

#### 'Community Link Evaluation'

A study specific questionnaire to assess service satisfaction, and utilisation of voluntary and community sector services. It contained 11-items on a Likert scale ranging from 1 to 4, 4 yes-no questions, and 7 open ended questions (available from the authors on request).

#### Primary Care Utilisation

Information on the number of GP and other primary care consultations, number of consultations that were about psychosocial problems, number of prescriptions of psychotropic medication, and of mental health related referrals (i.e. to psychiatrists, psychologists, counsellors, community metal health teams) were obtained from patient medical records for the three month period prior to the date of referral to the Community Link service and for the three month period following the patient's first appointment with the GPCMHW.

### Analysis

Data were analysed using the Statistical Package for the Social Sciences (version 11.5) [14]. The Kolmogrov-Smirnov test showed no evidence of departure from normality on the GHQ-12, CORE and WSAS; changes on these measures were analysed using t-tests for paired samples. The CSQ-8 and consultation data were not normally distributed and were analysed using non-parametric methods.

## Results

### Participants

**Figure 1:**
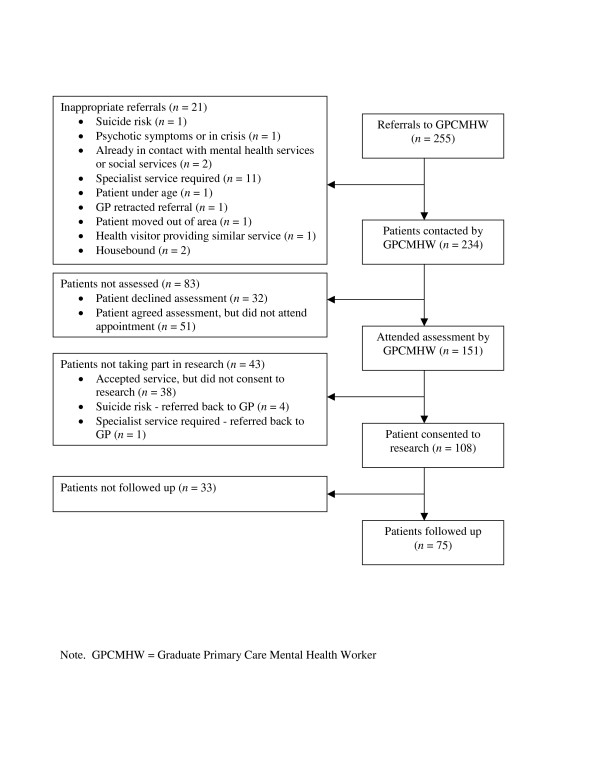
Consort diagram of patient flow through the Community Link Study.

Figure 1 shows the movement of patients through the study. Of the 146 patients who attended the assessment and were eligible, 108 consented to participate in the research study; 75/108 (69%) of these patients completed 3-month follow-up questionnaires.

Patients who consented were significantly more likely to speak English as a first language (82%) than patients who accessed the service but did not consent (60%) (X^2 ^= 6.038, d.f. = 1, *P *= 0.014). There were no other significant differences in demographics, presenting problems at referral, or baseline GHQ-12, CORE-OM or WSAS, between patients who consented and were successfully followed up and those who did not provide follow-up data.

Demographic data and presenting problems at referral (as assessed by the referring primary care team member) for the 108 patients are given in Table 1. Individuals were referred for a range of psychosocial problems. In relation to mental health, symptoms of depression were cited most frequently. The most common 'social' problem identified on referral forms was isolation.

**Table 1:** Demographics and clinical and social problems at referral

Variable	Value
Age, in years (n = 108)	Mean = 43.14 (SD = 14.56, range = 19–84)
Gender (n = 108)	*n *(%)
Male	41 (38.0)
Female	67 (62.0)
	
Ethnicity (n = 106)	
White (inc. White European)	71 (67.0)
Other	35 (33.0)
	
First language (n = 103)	
English	84 (81.6)
Other	19 (18.4)
	
Work status (n = 107)	
Employed	28 (26.2)
Unemployed	79 (73.8)
	
Benefits (n = 101)	
Yes	71 (70.3)
No	30 (29.7)
	
Clinical symptoms (n = 108)	
Depression	43 (39.8)
Anxiety	16 (14.8)
Mixed anxiety and depression	16 (14.8)
Other	16 (14.8)
None	17 (15.7)
	
Social problems (n = 108)	
Isolation	31 (28.7)
Personal relationships	18 (16.7)
Work	8 (7.4)
Welfare	8 (7.4)
Other	20 (18.5)
None	23 (21.3)

### Intervention

Nearly all patients were referred to the service by GPs. The mean waiting time to access the GPCMHW following referral was 22.18 (SD = 19.84) days. The modal number of patient appointments was 2 (range 1 – 3, with the exception of 4 patients, who were seen on 4 or more occasions). Information about community services was provided to 88% of patients, and the GPCMHW arranged to accompany 11 patients to the community services suggested.

### Client Satisfaction and Use of Suggested Services

The mean total score on the CSQ was 24.18 out of 32 points (SD = 5.54), which is considered moderate satisfaction [13]. Responses to specific items of the CSQ are given in Table 2. The mean of item 3 ('to what extent has our service met your needs?') was lower than for other items of the questionnaire (mean = 2.56, SD = 1.01). The response scale for this item is different to the other response scales, and the endorsement by patients was as follows: none of my needs have been met (16%), only a few of my needs have been met (34.7%), most of my needs have been met (26.7%), almost all of my needs have been met (22.6%).

**Table 2:** Patients' opinions of the Community Link service

Measure	Item	N	Negative %	Positive %
Client Satisfaction Questionnaire	How would you rate the quality of the service you have received?^a^	75	17.3	82.7
	Did you get the kind of service you wanted?^b^	75	30.7	69.3
	To what extent has our program met your needs?^c^	75	50.7	49.3
	If a friend were in need of similar help, would you recommend our program to him/her?^b^	75	10.7	89.3
	How satisfied are you with the amount of help you received?^d^	75	24.0	76.0
	Have the services you received helped you to deal more effectively with your problems?^e^	75	32.0	68.0
	In an overall, general sense, how satisfied are you with the service you received?^d^	75	22.7	77.3
	If you were to seek help again, would you come back to our program?^b^	75	21.3	78.7
Community Link Evaluation	Did your referrer give you enough information about the service?^b^	75	30.7	69.3
	Did the GPCMWH give you enough information about the service?^b^	74	2.7	97.3
	Did the GPCMHW understand the kind of support you wanted?^b^	73	12.3	87.7
	Did the GPCMHW suggest any services?^f^	72	0.0	100
	*If yes*, did the services the GPCMHW suggested match your interests?^b^	68	7.6	92.4
	Did you make use of the services?^f^	71	42.3	57.7
	*If yes*, are you still going?^f^	35	40	60
	*If yes*, were they relevant to your problem(s)?^b^	34	14.7	85.3
	*If yes*, did they help with your problem(s)?^b^	34	17.6	82.4
	Did you receive a telephone call from the GPCMHW 2–3 weeks after you last contact with them?^f^	65	4.6	95.4
	*If yes*, was it useful to hear from the GPCMHW?^b^	60	13.3	86.7
	Was the amount of support given by the GPCMHW about right?^b^	71	12.7	87.3
	Was the amount of contact you had with the GPCMHW about right?^b^	72	12.5	87.5
	Overall, do you feel better than you did before you saw the GPCMHW^b^	71	28.2	71.8
	Would you use the service again?^b^	67	10.4	89.6

^a^Answer categories: poor, fair (category: negative), good, excellent (category: positive)

^b^Answer categories: no, definitely not; no, not really (cat.: -); yes, generally; yes, definitely (cat.: +)

^c^Answer categories: none of my needs have been met, only a few of my needs have been met (cat.: -), most of my needs have been met, almost all of my needs have been met (cat.: +)

^d^Answer categories: quite dissatisfied, indifferent or mildly dissatisfied (cat.: -), mostly satisfied, very satisfied (cat.: +)

^e^Answer categories: no, they seemed to make things worse; no, they didn't help really (cat.: -); yes, they helped somewhat; yes, they helped a great deal (cat.: +)

^f^Answer categories: no (cat.: -), yes (cat.: +)

On the study-specific Community Link evaluation questionnaire (Table 2), over half of the patients (58%) reported accessing at least one of the services suggested and almost two-thirds of those were still attending. Most of the patients reported finding the services they accessed beneficial for their problems and most patients indicated they would use the Community Link service again.

Secondary analysis found that patients who had made contact with suggested community/voluntary services were more satisfied with the Community Link service (median CSQ = 26) than those who did not contact the services suggested (median CSQ = 22.83; U = 436.5, Z = -2.08, *P *= 0.037).

### Clinical and social outcomes

Table 3 gives data on clinical and social outcomes. On the GHQ-12, four-fifths of patients were cases at baseline (using the customary threshold of 2+), reducing to half post-intervention. There was a smaller reduction in the proportion of patients who were cases on the CORE-OM post-intervention. The clinical changes were accompanied by improvement in work and social adjustment scores on the WSAS.

**Table 3:** Pre- and post-intervention scores on the GHQ-12^1 ^CORE-OM^2 ^and WSAS^3^

Measure	*n*	Pre-intervention	Post-intervention	Difference (95% CI)
		Caseness		
		n (%)	n (%)	
GHQ-12	69	57 (82.6)	36 (52.2)	30.4% (16.9 – 43.9)
CORE-OM	74	63 (85.1)	50 (67.6)	17.5% (7.4 – 27.7)

		Mean (SD)	Mean (SD)	
GHQ-12	69	6.19 (4.04)	3.81 (4.40)	2.38 (1.25 – 3.51)
CORE-OM	74	17.7 (6.9)	15.0 (8.1)	2.7 (1.2 – 4.2)
WSAS	69	25.63 (11.86)	21.94 (12.95)	3.69 (1.54 – 5.84)

^1^General Health Questionnaire-12, ^2^Clinical Outcomes in Routine Evaluation – Outcomes Measure, ^3^Work and Social Adjustment Scale

### Primary Care Utilisation

There was a significant reduction in the recorded number of patient appointments (telephone and face-to-face) with GPs and other practice staff (z = 2.90, *P *= 0.003), in the mean number of consultations recorded as having a psychosocial aspect (z = 3.03, *P *= 0.002), and in the proportion of patients recorded to have been prescribed psychotropic medication, in the three months post intervention (see Table 4). There was, however, a significant increase in the proportion of patients who had a mental health related referral made on their behalf by the primary care team.

**Table 4:** Pre- and post-intervention resource use per patient (N = 101)

Variable	Pre-intervention	Post-intervention	Difference (95% CI)
	Median (range)	Median (range)	
PHC consultations	3 (1 – 14)	2 (0 – 13)	1 (1 – 2)
PHC consultations with a psychosocial aspect	1 (0 – 8)	0 (0 – 12)	1 (1 – 1)
			
	n (%)	n (%)	
Onwards MH related referrals	8 (7.9)	20 (19.8)	11.9% (1.9 – 21.9)
Psychotropic Medication	35 (34.7)	19 (18.8)	15.8% (6.0 – 25.6)

Note: PHC = Primary Health Care; MH = Mental Health

## Discussion

### Summary of Main Findings

Patients with a range of psychosocial presenting problems were helped to access voluntary and community organisations. Over half made contact with a community organisation identified as suitable for their needs and reported this to be beneficial for their problems. They were generally satisfied with the help provided by the GPCMHW in identifying and supporting access to a suitable service. There were significant reductions in psychological distress and improvements in work and social adjustment as measured by validated and reliable questionnaires. There were also significant reductions in the recorded number of consultations with the GP and other primary care team members and in the proportion of patients' prescribed psychotropic medication.

### Limitations of the Study

There are a number of limitations to the study. Firstly, the general practices involved volunteered for the study and may not be representative of practices overall. Secondly, only a proportion of patients referred were assessed and consented for the study and full follow-up data were not available on about a third. Thirdly, a longer follow-up period would have been desirable to allow patients time to engage fully and benefit more from the community organisations they contacted. Fourthly, as this was not a controlled study, only a tentative causal link between the intervention and the findings can be assumed.

### Comparisons with Existing Literature

The current study adds to evidence that primary care patients with psychosocial problems can be helped by facilitating their access to voluntary and community services. However, whilst previous studies have used workers who had extensive prior knowledge of local voluntary and community organisations [5-7], this study demonstrated that with appropriate support it was possible for graduates with limited training in mental health and no prior knowledge of community resources to carry out this role. This gives the possibility of much wider dissemination of such schemes.

Just under half the present sample did not make contact with the community organisation identified by the GPCMHW. This was despite the GPCMHW often contacting the organisation on the clients' behalf, and offering to support them in attending their initial meeting. Qualitative evaluations of other schemes have also commented on poor uptake of recommendations, but this is the first time this has been reported quantitatively [5,15]. Understanding why patients do not access recommended services, and adapting advice and support accordingly is likely to be very important in improving the impact of such schemes.

Whether patients acted on the recommendations and contacted local services or not the majority of patients felt that the service had met some (or more) of their needs. Whilst needing to be interpreted with caution, the finding that satisfaction was significantly greater for patients who contacted services implies that those services accessed were able to support the individuals' needs. Previous qualitative studies have also found patients to be generally satisfied [5,15].

## Conclusion

The finding that graduates with limited training in mental health and no prior knowledge of local community resources can help patients with psychosocial problems access voluntary and community services, gives the possibility of widespread adoption of such schemes. In the UK, this would be through GPCMHWs adding this to their roles within practices. This would also be consistent with a stepped care approach in primary care mental health, whereby briefer and less costly interventions are tried first, before more intensive mental health interventions [16-18].

However, while promising, the effectiveness of graduates in this role in reducing psychosocial and mental health problems needs to be confirmed in a randomised controlled trial. The randomised controlled trial of the Amalthea scheme in Bristol demonstrated that improved mental health outcomes were achieved using experienced local voluntary sector workers [7]. Future research needs to demonstrate this also for graduate workers without such experience.

## Funding and ethics approval

The study was funded by Camden and Islington Health Action Zone, Camden PCT and Islington PCT. The views expressed in this paper reflect those of the authors and not the funding bodies.

The study was reviewed by Camden and Islington Local Research Ethics Committee.

## Competing interests

The authors declare that they have no competing interests.

## Authors' contributions

JG collected and analysed data, participated in the interpretation of the data and was the joint main author of the manuscript. JC participated in the design of the study, interpretation of data, and was the joint main author of the manuscript. LO participated in the design and co-ordination of the study, interpretation of data, drafting of the manuscript. JL participated in the design and co-ordination of the study, interpretation of data, drafting of the manuscript. MB participated in the design of the study, interpretation of data, drafting of the manuscript. All authors read and approved the final manuscript.

## Pre-publication history

The pre-publication history for this paper can be accessed here:


